# Supplementary material for: Image steganography techniques for resisting statistical steganalysis attacks: A systematic literature review
Source: PLoS One. 2024 Sep 16;19(9):e0308807. doi: 10.1371/journal.pone.0308807 (PMC11404826; doi:10.1371/journal.pone.0308807)
Supplement: S1 Appendix — (DOCX) [file pone.0308807.s001.docx]

APPENDIX 1: DETAILED LIST OF REVIEWED STUDIES (RS)

[RS1] Al-Janabi, S., & Al-Shourbaji, I. (2016, December). A hybrid image steganography method based on genetic algorithm. In *2016 7th International Conference on Sciences of Electronics, Technologies of Information and Telecommunications (SETIT)*(pp. 398-404). IEEE.

[RS2] Baagyere, E. Y., Agbedemnab, P. A. N., Qin, Z., Daabo, M. I., & Qin, Z. (2020). A multi-layered data encryption and decryption scheme based on genetic algorithm and residual numbers. *IEEE Access*, *8*, 100438-100447.

[RS3] Sethi, P., & Kapoor, V. (2016). A proposed novel architecture for information hiding in image steganography by using genetic algorithm and cryptography. *Procedia Computer Science*, *87*, 61-66.

[RS4] Sabeti, V., Sobhani, M., & Hasheminejad, S. M. H. (2022). An adaptive image steganography method based on integer wavelet transform using genetic algorithm. *Computers and Electrical Engineering*, *99*, 107809.

[RS5] Shyla, M. K., Kumar, K. S., & Das, R. K. (2021). Image steganography using genetic algorithm for cover image selection and embedding. *Soft Computing Letters*, *3*, 100021.

[RS6] Ibanez, A. L., Djamal, E. C., Ilyas, R., & Najmurrokhman, A. (2018, July). Optimization of least significant bit steganography using genetic algorithm to improve data security. In *2018 10th International Conference on Information Technology and Electrical Engineering (ICITEE)* (pp. 523-528). IEEE.

[RS7] Joshi, S., & Sonawane, K. V. (2016, October). Selection of image blocks using genetic algorithm and effective embedding with DCT for steganography. In *Proceedings of the 9th Annual ACM India Conference* (pp. 161-166).

[RS8] Kanan, H. R., & Nazeri, B. (2014). A novel image steganography scheme with high embedding capacity and tunable visual image quality based on a genetic algorithm. *Expert systems with applications*, *41*(14), 6123-6130.

[RS9] Chandrasekaran, J., Arumugam, G., & Rajkumar, D. (2015, February). Ensemble of logistic maps with genetic algorithm for optimal pixel selection in image steganography. In *2015 2nd International Conference on Electronics and Communication Systems (ICECS)* (pp. 1172-1175). IEEE.

[RS10] Apau, R., Hayfron-Acquah, J. B., Asante, M., & Twum, F. (2023, August). A multilayered secure image steganography technique for resisting regular-singular steganalysis attacks using elliptic curve cryptography and genetic algorithm. In International Conference on ICT for Sustainable Development (pp. 427-439). Singapore: Springer Nature Singapore.

[RS11] Hu, D., Wang, L., Jiang, W., Zheng, S., & Li, B. (2018). A novel image steganography method via deep convolutional generative adversarial networks. *IEEE access*, *6*, 38303-38314.

[RS12] Yadav, G. S., Mangal, P., Parmar, G., & Soliya, S. (2023). Genetic algorithm and hamilton path based data hiding scheme including embedding cost optimization. Multimedia Tools and Applications, 82(13), 20233-20249.

[RS13] Bernard, S., Pevný, T., Bas, P., & Klein, J. (2019, July). Exploiting adversarial embeddings for better steganography. In *Proceedings of the ACM Workshop on Information Hiding and Multimedia Security* (pp. 216-221).

[RS14] Zhou, L., Feng, G., Shen, L., & Zhang, X. (2019). On security enhancement of steganography via generative adversarial image. *IEEE Signal Processing Letters*, *27*, 166-170.

[RS15] Li, L., Zhang, W., Qin, C., Chen, K., Zhou, W., & Yu, N. (2021). Adversarial batch image steganography against CNN-based pooled steganalysis. *Signal Processing*, *181*, 107920.

[RS16] Qin, C., Zhang, W., Dong, X., Zha, H., & Yu, N. (2021). Adversarial steganography based on sparse cover enhancement. *Journal of Visual Communication and Image Representation*, *80*, 103325.

[RS17] Sangeetha, K. N., Singh, S., Usha, B. A., & Ishaan Gonnagar, T. A. R. (2021, April). Security Enhancement in Image Steganography using Generative Adversarial Networks. In *2021 5th International Conference on Computing Methodologies and Communication (ICCMC)* (pp. 178-185). IEEE.

[RS18] Yadav, G. S. (2023). A genetic algorithm-based image steganography scheme with high embedding capacity and low distortion. The Imaging Science Journal, 69(1-4), 143-152.

[RS19] Liu, M., Luo, W., Zheng, P., & Huang, J. (2021). A new adversarial embedding method for enhancing image steganography. *IEEE Transactions on Information Forensics and Security*, *16*, 4621-4634.

[RS20] Liu, M., Song, T., Luo, W., Zheng, P., & Huang, J. (2022). Adversarial steganography embedding via stego generation and selection. *IEEE Transactions on Dependable and Secure Computing*.

[RS21] Zheng, Z., Hu, Y., Bin, Y., Xu, X., Yang, Y., & Shen, H. T. (2022). Composition-Aware Image Steganography Through Adversarial Self-Generated Supervision. *IEEE Transactions on Neural Networks and Learning Systems*.

[RS22] Shi, W., & Liu, S. (2022). Hiding message using a cycle generative adversarial network. *ACM Transactions on Multimedia Computing, Communications and Applications*, *18*(3s), 1-15.

[RS23] Wang, Z., Zhang, Z., & Jiang, J. (2021, October). Multi-Feature Fusion based Image Steganography using GAN. In *2021 IEEE International Symposium on Software Reliability Engineering Workshops (ISSREW)* (pp. 280-281). IEEE.

[RS24] Ubhi, J. S., & Aggarwal, A. K. (2022). Neural style transfer for image within images and conditional GANs for destylization. *Journal of Visual Communication and Image Representation*, *85*, 103483.

[RS25] Chen, X., Zhang, Z., Qiu, A., Xia, Z., & Xiong, N. N. (2020). Novel coverless steganography method based on image selection and stargan. *IEEE Transactions on Network Science and Engineering*, *9*(1), 219-230.

[RS26] Veerashetty, S. (2022). Secure communication over wireless sensor network using image steganography with generative adversarial networks. *Measurement: Sensors*, *24*, 100452.

[RS27] Saleema, A., & Amarunnishad, T. (2016). A new steganography algorithm using hybrid fuzzy neural networks. *Procedia Technology*, *24*, 1566-1574.

[RS28] Seethalakshmi, K. S., Usha, B. A., & Sangeetha, K. N. (2016, October). Security enhancement in image steganography using neural networks and visual cryptography. In *2016 International Conference on Computation System and Information Technology for Sustainable Solutions (CSITSS)*(pp. 396-403). IEEE.

[RS29] Jarusek, R., Volna, E., & Kotyrba, M. (2018). Robust steganographic method based on unconventional approach of neural networks. *Applied Soft Computing*, *67*, 505-518.

[RS30] Tang, W., Li, B., Tan, S., Barni, M., & Huang, J. (2019). CNN-based adversarial embedding for image steganography. *IEEE Transactions on Information Forensics and Security*, *14*(8), 2074-2087.

[RS31] Luo, T., Jiang, G., Yu, M., Zhong, C., Xu, H., & Pan, Z. (2019). Convolutional neural networks-based stereo image reversible data hiding method. *Journal of Visual Communication and Image Representation*, *61*, 61-73.

[RS32] Ashraf, Z., Roy, M. L., Muhuri, P. K., & Lohani, Q. D. (2020). Interval type-2 fuzzy logic system based similarity evaluation for image steganography. *Heliyon*, *6*(5).

[RS33] Dhawan, S., & Gupta, R. (2021). High‐quality steganography scheme using hybrid edge detector and Vernam algorithm based on hybrid fuzzy neural network. *Concurrency and Computation: Practice and Experience*, *33*(24), e6448.

[RS34] Srinivasu, L. N., & Veeramani, V. (2022). CNN based “Text in Image” Steganography using Slice Encryption Algorithm and LWT. *Optik*, *265*, 169398.

[RS35] Jain, T. (2020, February). Spatial domain steganography techniques and neural network based steganalysis with differential storage. In *2020 International Conference on Emerging Trends in Information Technology and Engineering (ic-ETITE)* (pp. 1-4). IEEE.

[RS36] Zhu, X., Lai, Z., Liang, Y., Xiong, J., & Wu, J. (2022). Generative high-capacity image hiding based on residual CNN in wavelet domain. *Applied Soft Computing*, *115*, 108170.

[RS37] Meng, L., Jiang, X., Zhang, Z., Li, Z., & Sun, T. (2022). A Robust Coverless Image Steganography Based on an End-to-End Hash Generation Model. *IEEE Transactions on Circuits and Systems for Video Technology*.

[RS38] Li, Z., Han, G., Guo, S., & Hu, C. (2019, August). Deepkeystego: Protecting communication by key-dependent steganography with deep networks. In *2019 IEEE 21st International Conference on High Performance Computing and Communications; IEEE 17th International Conference on Smart City; IEEE 5th International Conference on Data Science and Systems (HPCC/SmartCity/DSS)* (pp. 1937-1944). IEEE.

[RS39] Luo, Y., Qin, J., Xiang, X., & Tan, Y. (2020). Coverless image steganography based on multi-object recognition. *IEEE Transactions on Circuits and Systems for Video Technology*, *31*(7), 2779-2791.

[RS40] Tang, W., Li, B., Barni, M., Li, J., & Huang, J. (2020). An automatic cost learning framework for image steganography using deep reinforcement learning. *IEEE Transactions on Information Forensics and Security*, *16*, 952-967.

[RS41] Yadav, G. S., & Ojha, A. (2018). Hamiltonian path based image steganography scheme with improved imperceptibility and undetectability. *Applied Soft Computing*, *73*, 497-507.

[RS42] Iranpour, M. (2013, October). LSB-based steganography using Hamiltonian paths. In *2013 Ninth International Conference on Intelligent Information Hiding and Multimedia Signal Processing* (pp. 586-589). IEEE.

[RS43] Hong, W., Chen, T. S., & Luo, C. W. (2012). Data embedding using pixel value differencing and diamond encoding with multiple-base notational system. *Journal of Systems and Software*, *85*(5), 1166-1175.

[RS44] Lee, Y. P., Lee, J. C., Chen, W. K., Chang, K. C., Su, J., & Chang, C. P. (2012). High-payload image hiding with quality recovery using tri-way pixel-value differencing. *Information sciences*, *191*, 214-225.

[RS45] Zhan, W., Peng, Z., Hong, W., Chen, M., & Wen, D. (2015, December). A secure data hiding method based on patched reference table and pixel value differencing technique. In *2015 International Conference on Computational Intelligence and Communication Networks (CICN)* (pp. 1199-1202). IEEE.

[RS46] Shukla, A. K., Singh, A., Singh, B., & Kumar, A. (2018). A secure and high-capacity data-hiding method using compression, encryption and optimized pixel value differencing. *IEEE Access*, *6*, 51130-51139.

[RS47] Kaur, M., Kumar, V., & Singh, D. (2020). An efficient image steganography method using multiobjective differential evolution. In *Digital Media Steganography* (pp. 65-79). Academic Press.

[RS48] Chuang, Y. H., Lin, B. S., Chen, Y. X., & Shiu, H. J. (2021). Steganography in RGB images using adjacent mean. *IEEE access*, *9*, 164256-164274.

[RS49] Lee, J. H., Kang, D. Y., Lee, J. E., Lee, S. H., & Park, J. I. (2020, July). Automatic Recovery of Hidden Image from Image Steganography Using DNN and Local Entropy Features. In *2020 35th International Technical Conference on Circuits/Systems, Computers and Communications (ITC-CSCC)* (pp. 440-445). IEEE.

[RS50] Mandal, P. C., & Mukherjee, I. (2021, May). Integer Wavelet Transform based Secured Image Steganography using LSB and Coefficient Value Differencing. In *2021 2nd International Conference on Secure Cyber Computing and Communications (ICSCCC)* (pp. 332-337). IEEE.

[RS51] Chefranov, A. G., & Öz, G. (2022). Adaptive to pixel value and pixel value difference irreversible spatial data hiding method using modified LSB for grayscale images. *Journal of Information Security and Applications*, *70*, 103314.

[RS52] Walia, R. (2013, August). Steganography based on neighbourhood pixels. In *2013 International Conference on Advances in Computing, Communications and Informatics (ICACCI)* (pp. 203-206). IEEE.

[RS53] Alam, S., Kumar, V., Siddiqui, W. A., & Ahmad, M. (2014, February). Key dependent image steganography using edge detection. In *2014 Fourth International Conference on Advanced Computing & Communication Technologies* (pp. 85-88). IEEE.

[RS54] Islam, S., & Gupta, P. (2014, August). Robust edge based image steganography through pixel intensity adjustment. In *2014 IEEE Intl Conf on High Performance Computing and Communications, 2014 IEEE 6th Intl Symp on Cyberspace Safety and Security, 2014 IEEE 11th Intl Conf on Embedded Software and Syst (HPCC, CSS, ICESS)* (pp. 771-777).

[RS55] Sajasi, S., & Moghadam, A. M. E. (2015). An adaptive image steganographic scheme based on noise visibility function and an optimal chaotic based encryption method. *Applied Soft Computing*, *30*, 375-389.

[RS56] Al-Dmour, H., & Al-Ani, A. (2016). Quality optimized medical image information hiding algorithm that employs edge detection and data coding. *Computer methods and programs in biomedicine*, *127*, 24-43.

[RS57] Gaurav, K., & Ghanekar, U. (2018). Image steganography based on Canny edge detection, dilation operator and hybrid coding. *Journal of Information Security and Applications*, *41*, 41-51.

[RS58] Ismail, K. I. C. H., & Taouil, Y. (2018, December). Image steganography based on edge detection algorithm. In *2018 International Conference on Electronics, Control, Optimization and Computer Science (ICECOCS)* (pp. 1-4). IEEE.

[RS59] Hameed, M. A., Hassaballah, M., Aly, S., & Awad, A. I. (2019). An adaptive image steganography method based on histogram of oriented gradient and PVD-LSB techniques. *IEEE Access*, *7*, 185189-185204.

[RS60] Kumar, S., Singh, A., & Kumar, M. (2019). Information hiding with adaptive steganography based on novel fuzzy edge identification. *Defence Technology*, *15*(2), 162-169.

[RS61] Setiadi, D. R. I. M., Rustad, S., Andono, P. N., & Shidik, G. F. (2023). Graded fuzzy edge detection for imperceptibility optimization of image steganography. *The Imaging Science Journal*, 1-13.

[RS62] Lu, W., He, L., Yeung, Y., Xue, Y., Liu, H., & Feng, B. (2018). Secure binary image steganography based on fused distortion measurement. *IEEE Transactions on Circuits and Systems for Video Technology*, *29*(6), 1608-1618.

[RS63] Dhanasekaran, K., Anandan, P., & Kumaratharan, N. (2020). A robust image steganography using teaching learning based optimization based edge detection model for smart cities. *Computational Intelligence*, *36*(3), 1275-1289.

[RS64] Sultana, H., Kamal, A. H. M., Hossain, G., & Kabir, M. A. (2023). A novel hybrid edge detection and LBP Code-Based Robust Image Steganography method. Future Internet, 15(3), 108.

[RS65] Sarrafpour, B. A. S., Alomirah, R. A., Sarrafpour, S., & Sharifzadeh, H. (2021, October). An Adaptive Edge-Based Steganography Algorithm for Hiding Text into Images. In *2021 IEEE 19th International Conference on Embedded and Ubiquitous Computing (EUC)* (pp. 109-116). IEEE.

[RS66] Lou, D. C., & Hu, C. H. (2012). LSB steganographic method based on reversible histogram transformation function for resisting statistical steganalysis. *Information Sciences*, *188*, 346-358.

[RS67] Hong, W., Chen, T. S., & Wu, M. C. (2013). An improved human visual system based reversible data hiding method using adaptive histogram modification. *Optics Communications*, *291*, 87-97.

[RS68] Wang, J., Ni, J., & Zhang, X. (2016, June). Efficient HS based reversible data hiding using multi-feature complexity measure and optimized histogram. In *Proceedings of the 4th ACM Workshop on Information Hiding and Multimedia Security* (pp. 29-38).

[RS69] Musanna, F., & Kumar, S. (2020). Generating visually coherent encrypted images with reversible data hiding in wavelet domain by fusing chaos and pairing function. *Computer Communications*, *162*, 12-30.

[RS70] Xie, X. Z., Chang, C. C., & Lin, C. C. (2019). A hybrid reversible data hiding for multiple images with high embedding capacity. *IEEE Access*, *8*, 37-52.

[RS71] Xiong, X., Chen, Y., Fan, M., & Zhong, S. (2022). Adaptive reversible data hiding algorithm for interpolated images using sorting and coding. *Journal of Information Security and Applications*, *66*, 103137.

[RS72] Gao, K., Horng, J. H., & Chang, C. C. (2022). High-capacity reversible data hiding in encrypted images based on adaptive block encoding. *Journal of Visual Communication and Image Representation*, *84*, 103481.

[RS73] Wang, X., Chang, C. C., Lin, C. C., & Chang, C. C. (2022). Reversal of pixel rotation: A reversible data hiding system towards cybersecurity in encrypted images. *Journal of Visual Communication and Image Representation*, *82*, 103421.

[RS74] Singh, P. K., Jana, B., & Datta, K. (2022). Superpixel based robust reversible data hiding scheme exploiting Arnold transform with DCT and CA. *Journal of King Saud University-Computer and Information Sciences*, *34*(7), 4402-4420.

[RS75] Al-Omari, Z. Y., & Al-Taani, A. T. (2017, April). Secure LSB steganography for colored images using character-color mapping. In *2017 8th International Conference on Information and Communication Systems (ICICS)* (pp. 104-110).

[RS76] Chen, B., Luo, W., & Zheng, P. (2019, July). Enhancing steganography via stego post-processing by reducing image residual difference. In *Proceedings of the ACM Workshop on Information Hiding and Multimedia Security* (pp. 63-68).

[RS77] Song, T., Liu, M., Luo, W., & Zheng, P. (2021, June). Enhancing image steganography via stego generation and selection. In *ICASSP 2021-2021 IEEE International Conference on Acoustics, Speech and Signal Processing (ICASSP)* (pp. 2695-2699). IEEE.

[RS78] Li, F., Zeng, Y., Zhang, X., & Qin, C. (2022). Ensemble stego selection for enhancing image steganography. *IEEE Signal Processing Letters*, *29*, 702-706.

[RS79] Ding, X., Xie, Y., Li, P., Cui, M., & Chen, J. (2020). Image steganography based on artificial immune in mobile edge computing with internet of things. *IEEE Access*, *8*, 136186-136197.

[RS80] Raftari, N., & Moghadam, A. M. E. (2012, July). Digital image steganography based on assignment algorithm and combination of DCT-IWT. In *2012 Fourth International Conference on Computational Intelligence, Communication Systems and Networks* (pp. 295-300). IEEE.

[RS81] Narasimmalou, T., & Allen, J. R. (2012, August). Optimized discrete wavelet transform based steganography. In *2012 IEEE International Conference on Advanced Communication Control and Computing Technologies (ICACCCT)* (pp. 88-91). IEEE.

[RS82] Levický, D., Bugár, G., & Bánoci, V. (2012, September). A novel JPEG steganography method secure against histogram steganalysis. In *Proceedings ELMAR-2012* (pp. 79-82). IEEE.

[RS83] Bilal, M., Imtiaz, S., Abdul, W., & Ghouzali, S. (2013, May). Zero-steganography using DCT and spatial domain. In *2013 ACS international conference on computer systems and applications (AICCSA)* (pp. 1-7). IEEE.

[RS84] Lin, Y. K. (2014). A data hiding scheme based upon DCT coefficient modification. *Computer Standards & Interfaces*, *36*(5), 855-862.

[RS85] Attaby, A. A., Ahmed, M. F. M., & Alsammak, A. K. (2018). Data hiding inside JPEG images with high resistance to steganalysis using a novel technique: DCT-M3. *Ain Shams Engineering Journal*, *9*(4), 1965-1974.

[RS86] Zhang, X., Peng, F., & Long, M. (2018). Robust coverless image steganography based on DCT and LDA topic classification. *IEEE Transactions on Multimedia*, *20*(12), 3223-3238.

[RS87] Cogranne, R., Giboulot, Q., & Bas, P. (2021). Efficient steganography in JPEG images by minimizing performance of optimal detector. *IEEE Transactions on Information Forensics and Security*, *17*, 1328-1343.

[RS88] Qiao, T., Wang, S., Luo, X., & Zhu, Z. (2021). Robust steganography resisting JPEG compression by improving selection of cover element. *Signal Processing*, *183*, 108048.

[RS89] Tevaramani, S. S., & Ravi, J. (2022). Image steganography performance analysis using discrete wavelet transform and alpha blending for secure communication. *Global Transitions Proceedings*, *3*(1), 208-214.

[RS90] Bugár, G., Bánoci, V., Broda, M., Levický, D., & Dupák, D. (2014, April). Data hiding in still images based on blind algorithm of steganography. In *2014 24th International Conference Radioelektronika* (pp. 1-4). IEEE.

[RS91] Atta, R., & Ghanbari, M. (2018). A high payload steganography mechanism based on wavelet packet transformation and neutrosophic set. *Journal of visual communication and image representation*, *53*, 42-54.

[RS92] Zhang, H., & Hu, L. (2019). A data hiding scheme based on multidirectional line encoding and integer wavelet transform. *Signal Processing: Image Communication*, *78*, 331-344.

[RS93] Atta, R., Ghanbari, M., & IEEE, L. F. (2021). A high payload data hiding scheme based on dual tree complex wavelet transform. *Optik*, *226*, 165786.

[RS94] Mandal, P. C., Mukherjee, I., & Chatterji, B. N. (2021). High capacity steganography based on IWT using eight-way CVD and n-LSB ensuring secure communication. *Optik*, *247*, 167804.

[RS95] Ganguly, S., & Mukherjee, I. (2022, November). Image Sterilization through Adaptive Noise Blending in Integer Wavelet Transformation. In *2022 IEEE 19th India Council International Conference (INDICON)* (pp. 1-6). IEEE.

[RS96] Hassaballah, M., Hameed, M. A., Awad, A. I., & Muhammad, K. (2021). A novel image steganography method for industrial internet of things security. *IEEE Transactions on Industrial Informatics*, *17*(11), 7743-7751.

[RS97] Wang, C. C., Chang, Y. F., Chang, C. C., Jan, J. K., & Lin, C. C. (2014). A high capacity data hiding scheme for binary images based on block patterns. *Journal of Systems and Software*, *93*, 152-162.

[RS98] Al-Aidroos, N. M., & Bahamish, H. A. (2019, December). Image steganography based on LSB matching and image enlargement. In *2019 First International Conference of Intelligent Computing and Engineering (ICOICE)* (pp. 1-6).

[RS99] Tseng, H. W., & Leng, H. S. (2022). A reversible modified least significant bit (LSB) matching revisited method. *Signal Processing: Image Communication*, *101*, 116556.

[RS100] Sahu, A. K., & Swain, G. (2022). High fidelity based reversible data hiding using modified LSB matching and pixel difference. *Journal of King Saud University-Computer and Information Sciences*, *34*(4), 1395-1409.

[RS101] Huang, C. T., Wang, W. J., Tsai, M. Y., & Lee, C. F. (2012, September). Employing LSB and VQ for Undetectable Secret Data Hiding. In *2012 9th International Conference on Ubiquitous Intelligence and Computing and 9th International Conference on Autonomic and Trusted Computing* (pp. 644-649). IEEE.

[RS102] Sun, H., Luo, H., Wu, T. Y., & Obaidat, M. S. (2015, December). A psnr-controllable data hiding algorithm based on lsbs substitution. In *2015 IEEE Global Communications Conference (GLOBECOM)* (pp. 1-7). IEEE.

[RS103] Sapra, P. S., & Mittal, H. (2016, December). Secured LSB modification using dual randomness. In *2016 International Conference on Recent Advances and Innovations in Engineering (ICRAIE)* (pp. 1-4). IEEE.

[RS104] Tasheva, A., Tasheva, Z., & Nakov, P. (2017, June). Image based steganography using modified LSB insertion method with contrast stretching. In *Proceedings of the 18th International Conference on Computer Systems and Technologies* (pp. 233-240).

[RS105] Walia, G. S., Makhija, S., Singh, K., & Sharma, K. (2018). Robust stego-key directed LSB substitution scheme based upon cuckoo search and chaotic map. *Optik*, *170*, 106-124.

[RS106] Molato, M. R. D., Gerardo, B. D., & Medina, R. P. (2018, October). Secured data hiding and sharing using improved LSB-based image steganography technique. In *Proceedings of the 4th International Conference on Industrial and Business Engineering* (pp. 238-243).

[RS107] Deo, C. K., Singh, A., Singh, D. K., & Soni, N. K. (2020, July). Developing a Highly Secure and High Capacity LSB Steganography Technique using PRNG. In *2020 International Conference on Computational Performance Evaluation (ComPE)* (pp. 136-140). IEEE.

[RS108] C. Alipour, M., D. Gerardo, B., & P. Medina, R. (2020, December). LSB substitution image steganography based on randomized pixel selection and one-time pad encryption. In *Proceedings of the 2020 2nd International Conference on Big-data Service and Intelligent Computation* (pp. 1-6).

[RS109] Parmar, V., Gandhi, D., Srivastava, A., Swain, D., & Sharma, S. (2022, December). Efficient Data Hiding Method in Image Based on Modified LSB. In *2022 IEEE 2nd International Symposium on Sustainable Energy, Signal Processing and Cyber Security (iSSSC)* (pp. 1-6). IEEE.

[RS110] Duan, X., Li, B., Yin, Z., Zhang, X., & Luo, B. (2023). Robust image steganography against lossy JPEG compression based on embedding domain selection and adaptive error correction. Expert Systems with Applications, 229, 120416.

[RS111] Alanzy, M., Alomrani, R., Alqarni, B., & Almutairi, S. (2023). Image Steganography Using LSB and Hybrid Encryption Algorithms. Applied Sciences, 13(21), 11771.

[RS112] Sukumar, T., & Santha, K. R. (2015, March). An approach for secret communication using adaptive key technique for gray scale images. In *2015 International Conference on Circuits, Power and Computing Technologies [ICCPCT-2015]* (pp. 1-5). IEEE.

[RS113] Negi, L., & Negi, L. (2021, July). Hybrid approach for Data Security using Coverless Image Steganography with AES. In *2021 6th International Conference on Communication and Electronics Systems (ICCES)* (pp. 1077-1083). IEEE.

[RS114] Tiwari, K., & Gangurde, S. J. (2021, May). LSB steganography using pixel locator sequence with AES. In *2021 2nd International Conference on Secure Cyber Computing and Communications (ICSCCC)* (pp. 302-307). IEEE.

[RS115] Shanthakumari, R., Varadhaganapathy, S., Vinothkumar, S., & Bharaneeshwar, B. (2021, February). Data hiding in image steganography using range technique for secure communication. In *2021 International Conference on Advances in Electrical, Computing, Communication and Sustainable Technologies (ICAECT)* (pp. 1-7). IEEE.

[RS116] Wu, B., Xie, D., Chen, F., Wang, X., & Zeng, Y. (2022). A multi-party secure encryption-sharing hybrid scheme for image data base on compressed sensing. *Digital Signal Processing*, *123*, 103391.

[RS117] Maity, S. P., & Kundu, M. K. (2013). Distortion free image-in-image communication with implementation in FPGA. *AEU-International Journal of Electronics and Communications*, *67*(5), 438-447.

[RS118] Patwari, B., Nandi, U., & Ghosal, S. K. (2023). Image steganography based on difference of Gaussians edge detection. Multimedia Tools and Applications, 82(28), 43759-43779.

[RS119] Rahman, S., Uddin, J., Hussain, H., Ahmed, A., Khan, A. A., Zakarya, M., ... & Haleem, M. (2023). A Huffman code LSB based image steganography technique using multi-level encryption and achromatic component of an image. Scientific Reports, 13(1), 14183.

[RS120] Sedighi, V., Cogranne, R., & Fridrich, J. (2015). Content-adaptive steganography by minimizing statistical detectability. *IEEE Transactions on Information Forensics and Security*, *11*(2), 221-234.

[RS121] Kustov, V., & Silanteva, E. (2020, July). ±1Highly Undetectable Stegosystem Model Using Digital Still Images. In *2020 43rd International Conference on Telecommunications and Signal Processing (TSP)* (pp. 6-9). IEEE.

[RS122] Ansari, A. S., Mohammadi, M. S., & Parvez, M. T. (2020). A multiple-format steganography algorithm for color images. *IEEE Access*, *8*, 83926-83939.

[RS123] Liao, X., Yin, J., Chen, M., & Qin, Z. (2020). Adaptive payload distribution in multiple images steganography based on image texture features. *IEEE Transactions on Dependable and Secure Computing*, *19*(2), 897-911.

[RS124] Liu, X., Fang, Y., He, F., Li, Z., Zhang, Y., & Zeng, X. (2021, October). High capacity coverless image steganography method based on geometrically robust and chaotic encrypted image moment feature. In *2021 IEEE International Conference on Systems, Man, and Cybernetics (SMC)* (pp. 1455-1460). IEEE.

[RS125] Su, W., Ni, J., Hu, X., & Huang, F. (2022). Towards improving the security of image steganography via minimizing the spatial embedding impact. *Digital Signal Processing*, *131*, 103758.
