# Supplementary material for: Image steganography techniques for resisting statistical steganalysis attacks: A systematic literature review
Source: PLoS One. 2024 Sep 16;19(9):e0308807. doi: 10.1371/journal.pone.0308807 (PMC11404826; doi:10.1371/journal.pone.0308807)
Supplement: Attachment — Submitted filename: 2nd-comments.pdf [file pone.0308807.s005.pdf]

Title:

Image Steganography Techniques for Resisting Statistical Steganalysis Attacks: A Systematic Literature Review

Comments (Round #2):

Authors have revised and completed the manuscript according to my review from the previous round, especially to emphasize the RQ2 in their surveys.

The discussion about RQ2 is now more complete with an exploration of methods along with the relevant problems. Additional information in Tables 7, 8, and 9 as well as the given brief explanation can enrich the reader's knowledge, especially on research topics in image hiding and steganography fields.

Decision: Accepted

Reviewer,

Aris Marjuni
