# Supplementary material for: Image steganography techniques for resisting statistical steganalysis attacks: A systematic literature review
Source: PLoS One. 2024 Sep 16;19(9):e0308807. doi: 10.1371/journal.pone.0308807 (PMC11404826; doi:10.1371/journal.pone.0308807)
Supplement: Attachment — Submitted filename: Second Rebuttal Letter to Editor.docx [file pone.0308807.s006.docx]

Once again, We want would like to thank the Academic Editor, Associate Editor , the three anonymous Reviewers of the revised manuscript and the entire team at PLOS ONE Journal. We much appreciate the efforts that have been put in place to ensure the richness and the much quality of our article now. we are grateful and look forward for final acceptance.

We also took note of the additional comments from the Editor. This letter provides response to the comments raised. Where the comments have been resolved it is indicated, where the comments were not, reasonable explanations have been provided.

Thank you.

**Editor Comments:**

**Comment 1**: Authors should reflect the importance of the article in the abstract.

**Response:** We have included in the abstract the main aim of the article and its importance to image steganography researchers and data protection practitioners.

**Comment 2:** The abstract is too long, please review and shorten it.

**Response:** This has been addressed. We have reviewed and shortened the abstract from the initial 300 words to 250 words.

**Comment 3:** Although 125 references are also mentioned in the abstract, there are 147 items in the references section.

**Response:** There is a difference between the 125 articles referenced in the abstract and the reference list generated at the end of the article. The 125 mentioned in the abstract is the total number of articles used to perform the systematic literature review. All those articles have been added at the end as APPENDIX I (RS1-RS125). This is separate from works cited in the paper which is the reference list of 148.

**Comment 4:** Use more academic language, have the article checked for grammar.

**Response:** we have performed academic language review and corrected where necessary. We have also used software including Grammarly to correct identified grammatical errors.

**Comment 5**: Check your section numbers.

**Response:** Ce have checked our section numbers. They are all sequential and correctly numbered.

**Comment 6:** Review your tables, figures and equations again.

**Response:** Table, figures and equations have been reviewed again to ensure they are correctly labelled and titled.

**Comment 7:** You should increase the figure quality.

**Response:** Figures whose quality was deemed low have been regenerated to ensure high resolution.

**Comment 8:** Check your fonts and size.

**Response:** Font size throughout the article have been set to **times new roman 11** as per **PLOS ONE specifications**, except in tables where a lower font size is chosen to fit the words.

**Comments by REVIEWERS:**

We noticed that all reviewers accepted the revised manuscript as fully meeting their review requirements without further review. All reviewers appreciated the revised manuscript.
